# Lymphocyte-C-reactive protein ratio with calf circumference could better predict survival of patients with non-metastatic cancer

**DOI:** 10.1038/s41598-023-34096-w

**Published:** 2023-05-03

**Authors:** Xiao-Yue Liu, Xi Zhang, Qi Zhang, Guo-Tian Ruan, Hai-Lun Xie, Tong Liu, Meng-Meng Song, Yi-Zhong Ge, Li Deng, Han-Ping Shi

**Affiliations:** 1 Departments of Gastrointestinal Surgery and Clinical Nutrition, Beijing Shijitan Hospital, Capital Medical University, Beijing, 100038 China; 2 Beijing International Science and Technology Cooperation Base for Cancer Metabolism and Nutrition, Beijing, China; 3 Key Laboratory of Cancer FSMP for State Market Regulation, Beijing, China; 4 Department of Radiotherapy, Affiliated Hospital of Hebei University, Baoding, China

**Keywords:** Cancer, Biomarkers, Oncology

## Abstract

Systemic inflammatory responses caused by tumor cells play an important role in the occurrence and development of tumors. The aim of this study was to identify biomarkers that most accurately predict prognoses in patients with non-metastatic cancer and to evaluate their clinical significance when combined with muscle markers. This study retrospectively evaluated 2,797 cancer patients diagnosed with cancer at TNM stages I, II, and III. Lymphocyte-C-reactive protein ratio (LCR) in conjunction with calf circumference (CC) were used (or chosed) after evaluating the predictive value of 13 inflammatory marker combinations and five anthropometric indicators for patient outcomes using the C-index. The Kaplan–Meier method and Cox’s proportional hazards regression modeling were used to analyze the individual and combined effects of these two potential biomarkers on overall survival. This study enrolled 1,604 men (57.3%) and 1,193 women (42.7%) with a mean age of 58.75 years. Among the 13 inflammatory nutritional indicators, the LCR was the most accurate predictor of prognoses in patients with non-metastatic cancer. After multifactorial adjustment, we found that low LCR had an adverse effect on overall survival (hazard ratio [HR]: 2.50; 95% confidence interval [CI]: 2.17, 2.88; P < 0.001). Low LCR combined with low CC was also shown to be an independent risk factor for poor overall survival (HR: 2.26; 95% CI: 1.80, 2.83; P < 0.001). Compared with LCR or CC alone, the combination of the two had greater prognostic value for patients with non-metastatic cancer. The LCR can be implemented as a useful biomarker to predict prognoses in patients with non-metastatic cancer. CC is the best anthropometric indicator of muscle loss in patients with non-metastatic cancer. The combination of LCR and CC can better predict the prognosis of patients with non-metastatic cancer, and can provide important information for clinicians to formulate diagnosis and treatment plans.

## Introduction

Cancer is the leading cause of death in all countries worldwide, imposing a severe health and economic burden^1^. The number of cancer cases and deaths is growing rapidly given a growing and aging population and an increase in the prevalence of lifestyle risk factors^2^. As in the rest of the world, cancer has become a serious public health problem in China that has been attracting increasing attention^3^. Although cancer treatments have become more diversified in recent years, prognoses for many cancer patients remain poor. Generally, patients with early non-metastatic cancer have improved prognoses and longer survival times. Cancer patients with more complications are more likely to present with a high inflammatory state accompanied by muscle loss^4^. Therefore, it is important to identify more accurate and valuable prognostic parameters for patients with non-metastatic cancers. This could help clinicians identify problems and intervene earlier for patients with early-stage cancer.

Inflammation has become a recognized hallmark of cancer progression, leading to a series of cancer-associated symptoms, including fever, sweating, and weight loss^5^. The interaction of inflammation with the host tumor is now considered the seventh hallmark of cancer^6^. After tumor cells enter the body's blood circulation, they activate a series of inflammatory responses and stimulate the release of inflammatory factors and immune cells, thereby promoting cancer development^7^. Currently, some serological indicators are used to reflect the body's inflammation and nutritional status, and studies have shown that the prognostic nutritional index (PNI)^8^, the modified Glasgow prognostic score (mGPS)^9^, the lymphocyte-c-reactive protein ratio score (LCS)^10^, the geriatric nutritional risk index (GNRI)^11^, the nutritional risk index (NRI)^12^, the neutrophil to lymphocyte ratio (NLR)^13^, the platelet to lymphocyte ratio (PLR)^14^, the glucose to lymphocyte ratio (GLR)^15^, the advanced lung cancer inflammation index (ALI)^16^, the Systemic Immune-Inflammation Index (SII)^17^, the controlling nutritional status score (CONUT score)^18^, the lymphocyte-to-C-reactive protein ratio (LCR)^19^, the albumin–globulin ratio (AGR)^20^, and other inflammatory nutrition-associated indicators can be used as independent prognostic factors in cancer patients. Studies have shown that inflammation can promote the loss of muscle mass as well as decrease strength and muscle function in cancer patients^21^. Reductions of skeletal muscle in cancer patients indicate an increase in the toxic effects of cancer treatments^22^ and are associated with a decrease in survival rates^23^. Therefore, assessing the inflammatory state combined with muscle indicators in patients with non-metastatic cancer may predict prognoses effectively. Although the prognostic value of inflammatory nutritional indicators in cancer patients has been reported in prior studies, to the best of our knowledge, few studies have examined whether systemic inflammation and muscle mass predict prognoses in patients with non-metastatic cancer.

Hence, this study aimed to explore the best indicators for predicting prognoses for non-metastatic cancers. We evaluated 13 inflammatory nutrition-associated indicators and identified commonly used anthropometric indicators that were most valuable for predicting patient outcomes in 2,797 patients diagnosed with stage I-III cancer. We then assessed the independent effects as well as the joint associations between these indicators and patient survival.

## Methods

### Study population

**Figure 1:**
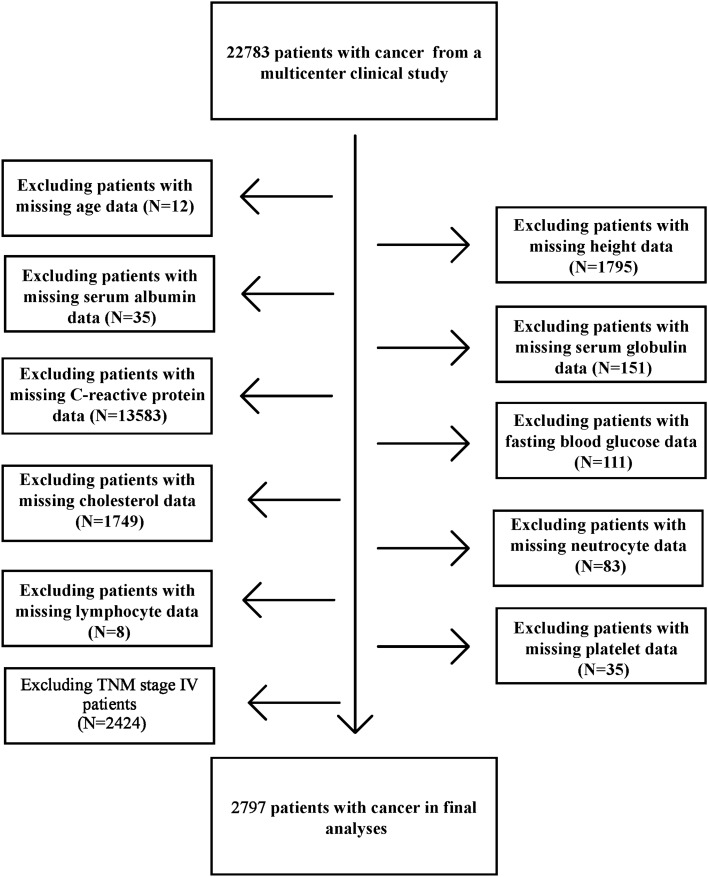
Flow chart.

This multicenter observational study investigated nutritional status and clinical results within the Chinese Common Cancer (INSCOC) cohort (registration number: ChiCTR1800020329; http://www.chictr.org.cn) from May 2013 to June 2021^24^. This prospective cohort collects data from multiple centers in China; the study design, methods, and study development process have been described earlier^25^. All patients enrolled in the INSCOC cohort were aged 18 years or older, were diagnosed with solid tumors, and received surgery, chemotherapy, radiotherapy, or other anti-cancer treatments; we enrolled hospitalized patients with a length of stay > 48 h. All patients included in the study were diagnosed with oncology at the time of their first hospitalization from May 2013 to June 2021. If patients experienced multiple hospitalizations, only the data from the first hospitalization were used for analysis. Patients with clinical evidence of active infection, patients presenting with immune disease, and patients lacking specific data on age, height, albumin levels, globulin levels, cholesterol levels, C-reactive protein (CRP) levels, blood glucose levels, neutrophil counts, lymphocyte counts, and platelets (PLT) counts were excluded from the current study. In addition, we excluded patients with TNM stage IV and those who were lost to follow-up during the follow-up process. The study followed the principles outlined in the Declaration of Helsinki and was approved by the ethics committees of all local study centers. Written informed consent for the use clinical data (without disclosing personal information) was obtained from all participants. Figure 1 shows a flow chart for the research object screening process.

### Patient and public involvement

Patients and members of the public were not involved in the design, conduct, reporting or dissemination of this research as it was an observational study focusing on the incidence of insulin resistance and inflammation and prognostic factors in women with cancer of the reproductive system.

### Patient characteristics

Patient age, sex, primary tumor type, tumor stage, smoking history, and drinking history were obtained from the electronic medical record system at the participating medical centers. Body mass index (BMI) was calculated for all patients, and was defined as weight (kg) divided by height (m) squared. Patients were divided into three groups: underweight (< 18.5 kg/m^2^) and normal weight (≥ 18.5 kg/m^2^). Clinical staging was evaluated based on the TNM staging system delineated in the 8th edition of the American Joint Committee on Cancer (AJCC) TNM staging system. Patient-generated subjective nutritional assessments (PG-SGA) and Karnofsky performance status (KPS) were assessed and recorded at baseline by trained staff. Serum albumin levels, CRP levels, hemoglobin (Hb) levels, PLT levels, and other serological indicators were obtained after the patients fasted overnight within 24 h following admission and were analyzed and standardized in the central laboratory to eliminate differences caused by different laboratory equipment.

### Inflammatory nutrition and muscle state

Patients’ inflammatory nutritional status (based on PNI, mGPS, LCS, GNRI, NRI, NLR, PLR, GLR, ALI, SII, CONUT score, LCR, and AGR readings) were reassessed according to data collected at baseline. The calculation formula for each index is shown in Supplementary Table 1.

In addition, anthropometric indicators were measured for all patients. Calf circumference (CC) was measured with a standard tape measure in 0.1 cm increments with knees bent to 90 degrees and relaxed feet and ankles. Mid-arm circumference (MAC) and triceps skinfold thickness (TSF) were measured in 0.1 cm and 1 mm increments, respectively. MAC was measured with a plastic meter, and TSF was measured with a skinfold caliper. Mid-arm muscle circumference (MAMC) was calculated using the following formula: MAMC (mm) = MAC (mm)—[3.14 × TSF (mm)]. Hand grip strength (HGS) was measured using a Jamar dynamometer to evaluate the strength of the patient’s non-dominant hand. The patients were asked to recall their weight six months prior and to compare it with their weight as measured at admission. We classified each index using maximally selected rank statistics to obtain the optimal cut-off point values.

### Outcomes

The primary endpoint of this study was all-cause mortality. The overall survival (OS) period was defined as the time from the date of admission to death or the last follow-up. Secondary endpoint events included length of stay (LOS), cost, and Karnofsky scores (KPS; a self-scoring of health status with a total score of 100 points and 10 points per level).

### Statistical analysis

Demographic characteristics of the study population were calculated, with continuous variables expressed as either means ± standard deviations or medians and interquartile ranges (IQR). Categorical variables were presented as numbers and percentages (n, %). Comparisons of differences between groups were conducted via independent Student’s t-tests or non-parametric tests for comparing continuous variables and via Chi-square tests or Fisher’s exact tests for comparing categorical variables. The optimal cut-off point values for all inflammatory nutritional indicators were obtained using maximally selected rank statistics. We selected covariates and potential confounders based on previous knowledge. Univariate and multivariate Cox regression analyses were used to evaluate hazard ratios (HRs) and 95% confidence intervals (CIs) for important prognostic factors based on OS. A sensitivity analysis excluding patients who died within six months of enrollment was performed. Kaplan–Meier (K-M) curves and log-rank tests were presented to evaluate time-patient survival trends and to compare survival between groups. The Harrell C index and the area under time-dependent curve (AUC) were calculated to evaluate and compare the predictive ability of inflammatory nutritional and anthropometric indicators for patient survival. Differences were considered statistically significant given two-sided *P* values of < 0.05. All statistical analyses were performed using R software, version 4.1.1 (The R Project for Statistical Computing, Vienna, Austria).

### Ethics approval and consent to participate

The study followed the principles outlined in the Declaration of Helsinki and was approved by the Medical Ethics Committee of Beijing Shijitan Hospital, Capital Medical University. Written informed consent was obtained from all the participants to use clinical data without disclosing personal information.

## Results

### Predicting prognoses in patients with non-metastatic cancer

When calculating the C-index (Supplementary Table 2) and AUC (Supplementary Fig. 1) for 13 inflammatory nutritional indicators and five anthropometric indicators, we found that the LCR (C-index = 0.65252) was the strongest predictor of survival in patients with non-metastatic cancer, although almost all research indicators statistically significantly predicted OS (Supplementary Table 3). The anthropometric index with the strongest prognostic ability was CC; this result was verified by evaluating the corresponding the time-dependent receiver operating characteristic (ROC) curve (Supplementary Fig. 2). Based on these findings, we decided to focus the analyses of this study on LCR in order to comprehensively evaluate its clinical impact and potential as a prognostic biomarker.

### Patient characteristics

A total of 2,797 cancer patients diagnosed with TNM stages I-III were enrolled in the current study. We enrolled 1,604 men (57.3%) and 1,193 women (42.7%), presenting with a mean age of 58.75 years. We classified each index using maximally selected rank statistics to obtain the optimal cut-off point values (Supplementary Fig. 3). Table 1 shows the characteristics of the 2,797 enrolled patients with non-metastatic cancer, classified according to LCR values. Compared with the high LCR group (> 2,500), patients in the low LCR group (≤ 2,500) were more likely to be male, elderly, and to present with lung cancer diagnoses, low BMI, poor tumor stage. These patients were also more likely to have low albumin levels, low Hb levels, high CRP levels, high PLT levels, and high PG-SGA scores.

**Table 1:** The baseline clinicopathological characteristics of 2797 patients with non-metastatic tumor according to LCR.

Characteristics	LCR		
	> 2500(n = 1929)	≤ 2500(n = 868)	*P* value
Age, year	57.79 (11.07)	60.91 (10.80)	< 0.001
Gender, n (%)			< 0.001
Male	1050 (54.4)	554 (63.8)	
Female	879 (45.6)	314 (36.2)	
BMI, kg/m^2^	22.84 (20.55, 25.06)	22.38 (19.72, 24.74)	< 0.001
Tumor stage, n (%)			< 0.001
I	340 (17.6)	101 (11.6)	
II	676 (35.0)	267 (30.8)	
III	913 (47.3)	500 (57.6)	
Tumor type, n (%)			< 0.001
Upper gastrointestinal cancer	437 (22.7)	211 (24.3)	
Colorectal cancer	383 (19.9)	136 (15.7)	
Lung cancer	516 (26.7)	339 (39.1)	
Breast cancer	291 (15.1)	37 ( 4.3)	
Hepatobiliary and pancreatic tumors	113 (5.9)	53 ( 6.1)	
Others	189 (9.8)	92 (10.6)	
Surgery, n (%)			< 0.001
No	687 (35.6)	446 (51.4)	
Yes	1242 (64.4)	422 (48.6)	
Chemotherapy, n (%)			0.422
No	688 (35.7)	324 (37.3)	
Yes	1241 (64.3)	544 (62.7)	
Radiotherapy, n (%)			0.033
No	1758 (91.1)	768 (88.5)	
Yes	171 (8.9)	100 (11.5)	
KPS			< 0.001
> 70	1822 (94.5)	718 (82.7)	
≤ 70	107 (5.5)	150 (17.3)	
Alb, g/L	41.00 (38.40, 43.70)	37.50 (34.10, 41.10)	< 0.001
CRP, g/L	2.97 (0.98, 3.23)	18.50 (9.30, 40.52)	< 0.001
Hb, g/L	130.00 (118.00, 142.00)	121.00 (103.00, 133.00)	< 0.001
PLT	214.00 (170.00, 264.00)	231.00 (172.00, 302.00)	< 0.001
NLR	1.88 (1.35, 2.60)	3.27 (2.13, 5.21)	< 0.001
PLR	126.47 (96.46, 168.42)	176.32 (121.11, 251.38)	< 0.001
GLR	3.22 (2.56, 4.27)	4.12 (3.09, 6.32)	< 0.001
ALI	50.09 (35.37, 69.85)	26.30 (14.89, 40.32)	< 0.001
SII	385.53 (260.13, 615.09)	768.90 (432.35, 1293.04)	< 0.001
CONUT, n (%)			< 0.001
≤ 1	1129 (58.5)	234 (27.0)	
> 1	800 (41.5)	634 (73.0)	
mGPS, n (%)			< 0.001
0	1922 (99.6)	247 (28.5)	
1	6 (0.3)	413 (47.6)	
2	1 (0.1)	208 (24.0)	
GNRI	101.26 (96.46, 105.58)	95.44 (88.60, 101.41)	< 0.001
AGR	1.45 (1.28, 1.66)	1.26 (1.06, 1.46)	< 0.001
PNI	49.70 (46.25, 53.10)	44.70 (40.20, 48.41)	< 0.001
NRI	102.46 (97.60, 106.87)	96.58 (89.70, 102.61)	< 0.001
LCS, n (%)			< 0.001
0	872 (45.2)	0 (0.0)	
1	1030 (53.4)	641 (73.8)	
2	27 (1.4)	227 (26.2)	
PGSGA, n (%)			< 0.001
0–3	980 (50.8)	281 (32.4)	
4–9	726 (37.6)	347 (40.0)	
> 9	223 (11.6)	240 (27.6)	
LCR	6646.34 (4451.22, 14,788.73)	751.84 (306.43, 1460.77)	< 0.001
HGS, kg	24.80 (18.97, 31.80)	24.10 (17.67, 30.92)	0.003
MAC, cm	27.00 (25.00, 28.70)	26.00 (24.00, 28.00)	< 0.001
MAMC, cm	21.69 (19.89, 23.58)	21.35 (19.49, 23.36)	0.006
CC, cm	34.00 (31.50, 36.30)	33.00 (30.50, 35.82)	< 0.001
TSF, mm	16.00 (10.00, 21.00)	14.00 (10.00, 20.00)	< 0.001

BMI, body mass index; Alb, albumin; CRP, C-reactive protein; Hb, hemoglobin; PLT, blood platelet; NLR, neutrophil-to-lymphocyte ratio; PLR, platelet-to-lymphocyte ratio; GLR, glucose-to-lymphocyte ratio; ALI, advanced lung cancer inflammation index; SII, systemic immune-inflammation index; CONUT, controlling nutritional status score; mGPS, modified Glasgow Prognostic Score; GNRI, Geriatric Nutritional Risk Index; AGR, albumin-globulin ratio; PNI, prognostic nutritional index; NRI, nutritional risk index; LCS, lymphocyte C-reactive protein score; LCR, lymphocyte-to-C-reactive protein (CRP) ratio; HGS, hand grip strength; MAC, mid-arm circumference; MAMC, mid-arm muscle circumference; CC, calf circumference; TSF, triceps skinfold.

### LCR, CC, and OS

**Figure 2:**
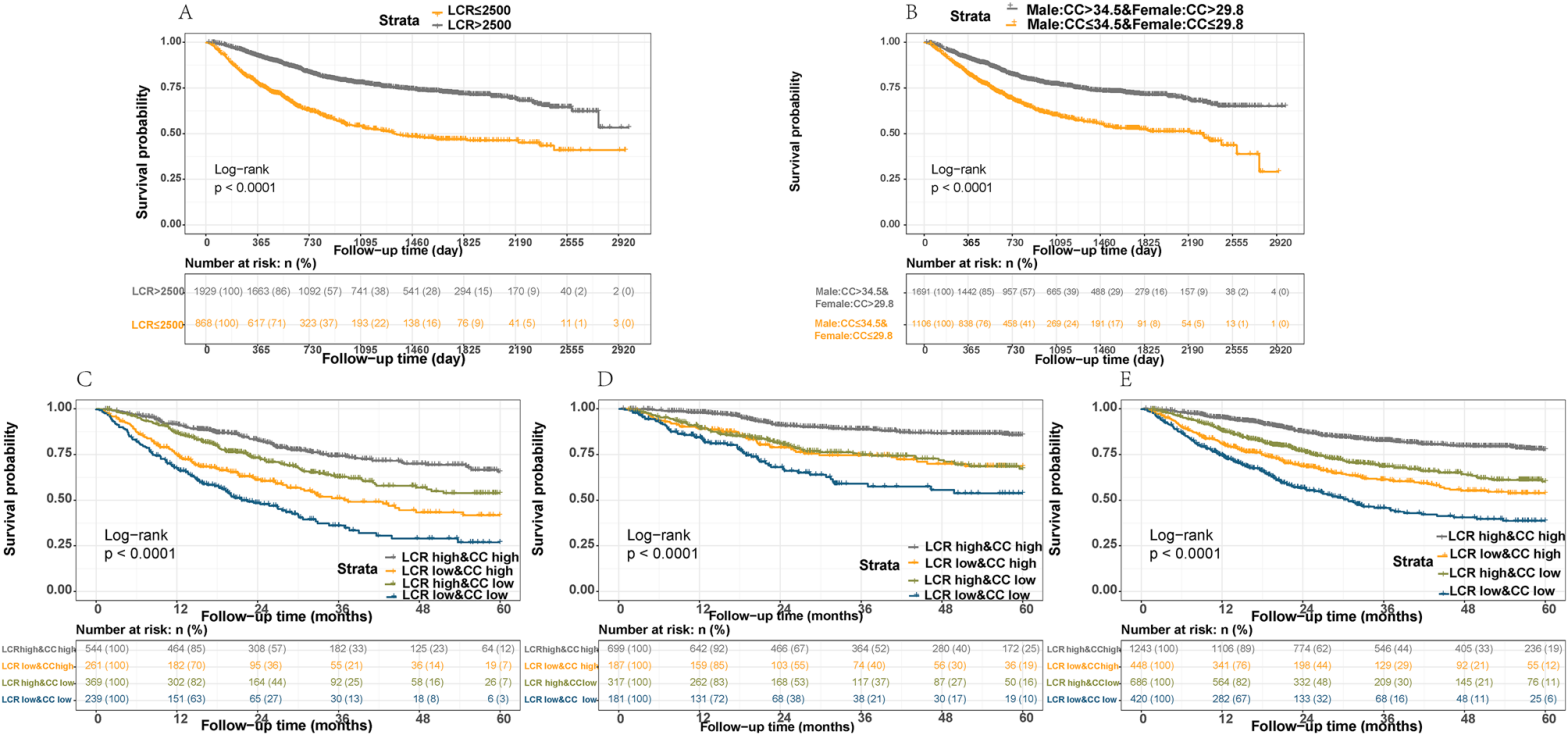
Survival curves via Kaplan–Meier analysis of LCR and CC. (**A**) Survival curves via Kaplan–Meier analysis of LCR. (**B**) Survival curves via Kaplan–Meier analysis of CC. (**C**) Survival curves for total tumor patients with LCR and CC. (**D**) Survival curves of TNM stage I and II tumor patients with LCR and CC E. Survival curves of TNM stage III tumor patients with LCR and CC.

We found that the LCR was negatively correlated with OS, regardless of whether the LCR was analyzed as a continuous (Fig. 2A; Supplementary Fig. 4A) or categorical variable (Supplementary Fig. 4B). Patients with a lower LCR tended to have poorer prognoses. In various adjusted models, we demonstrated that patients’ OS consistently improved with an increase in the LCR. This conclusion remained true even after patients with non-metastatic cancer were divided into early (stage I, II) and stage III cancer patients in secondary analyses (Table 2).

**Table 2:** Cox proportional analysis of LCR predicts OS in patients with non-metastatic tumors by different tumor stages.

	crude HR(95% CI)	P-value	adjusted HR (95% CI) ^a^	P-value	adjusted HR (95% CI) ^b^	P-value
Non-metastatic tumors						
LCR, as continuous	0.73 (0.64,0.83)	< 0.001	0.8 (0.71,0.90)	< 0.001	0.83 (0.74,0.93)	0.001
> 2500	Ref		Ref		Ref	
≤ 2500	2.50 (2.17,2.88)	< 0.001	2.14 (1.85,2.47)	< 0.001	1.74 (1.50,2.03)	< 0.001
< 1685	Ref		Ref		Ref	
1685–4696	0.61 (0.51,0.73)	< 0.001	0.65 (0.54,0.78)	< 0.001	0.78 (0.65,0.94)	0.008
4696–9355	0.35 (0.28,0.43)	< 0.001	0.41 (0.33,0.51)	< 0.001	0.52 (0.42,0.65)	< 0.001
> 9355	0.32 (0.26,0.39)	< 0.001	0.41 (0.33,0.50)	< 0.001	0.50 (0.40,0.61)	< 0.001
p for trend		< 0.001		< 0.001		< 0.001
TNM stage I and II tumors						
LCR, as continuous	0.73 (0.59,0.90)	0.004	0.78 (0.64,0.96)	0.017	0.78 (0.64,0.94)	0.011
> 2500	Ref		Ref		Ref	
≤ 2500	2.28 (1.78,2.91)	< 0.001	1.84 (1.43,2.37)	< 0.001	1.57 (1.2,2.05)	0.001
< 1685	Ref		Ref		Ref	
1685–4696	0.70 (0.51,0.96)	0.027	0.83 (0.60,1.14)	0.246	0.96 (0.69,1.33)	0.8
4696–9355	0.36 (0.25,0.52)	< 0.001	0.45 (0.31,0.65)	< 0.001	0.55 (0.37,0.80)	0.002
> 9355	0.33 (0.24,0.46)	< 0.001	0.43 (0.31,0.61)	< 0.001	0.49 (0.35,0.70)	< 0.001
p for trend		< 0.001		< 0.001		< 0.001
TNM stage III tumors						
LCR, as continuous	0.77 (0.66,0.89)	< 0.001	0.79 (0.69,0.92)	0.002	0.84 (0.73,0.96)	0.012
> 2500	Ref		Ref		Ref	
≤ 2500	2.41 (2.02,2.87)	< 0.001	2.29 (1.92,2.74)	< 0.001	1.80 (1.50,2.17)	< 0.001
< 1685	Ref		Ref		Ref	
1685–4696	0.57 (0.46,0.71)	< 0.001	0.58 (0.47,0.73)	< 0.001	0.72 (0.57,0.90)	0.004
4696–9355	0.37 (0.28,0.48)	< 0.001	0.40 (0.30,0.51)	< 0.001	0.53 (0.41,0.69)	< 0.001
> 9355	0.37 (0.29,0.48)	< 0.001	0.40 (0.31,0.52)	< 0.001	0.51 (0.39,0.67)	< 0.001
p for trend		< 0.001		< 0.001		< 0.001

a: Adjusted by age, sex, tumor stage, BMI; b: Adjusted for age, sex, tumor type, tumor stage, BMI, KPS, PG-SGA, surgery, radiotherapy, chemotherapy, smoking, drinking; LCR, lymphocyte-to-C-reactive protein (CRP) ratio; HR, hazard ratio; CI, confidence interval.

We evaluated maximally selected rank statistics to determine the optimal cut-off values for gender classifications with regard to CC (male: 34.5 cm; female: 29.8 cm). Patients with high CC values had improved prognoses as compared with those with low CC in various adjusted models (Fig. 2B; Supplementary Table 4).

### Predictions of OS

In survival analyses, we cross-classified the LCR and CC into four categories (high, high; low, low; high, low; and low, high). We calculated the mean survival time (in months), assessed K-M survival curves and performed Cox proportional survival analysis in different groups. In a multivariate adjusted Cox proportional risk model, we examined the combined effects of LCR and CC as independent predictors of survival.

It was found that in the group with high LCR combined high CC, the mean survival time was 52.12 (95% CI: 51.11–53.13) months, followed by the group with high LCR and low CC, and the group with low LCR and high CC, with 45.32 (95% CI: 43.58–47.06) and 41.17 (95% CI: 38.86–43.48) months, respectively. In contrast, the mean survival time in the group with both low was only 34.15 (95% CI: 31.58–36.72) months, and the difference was statistically significant (Supplementary Table 5, P < 0.001). In addition, we calculated the mean survival time in different subgroups and the results were consistent with the total population and the differences were significant through continuing significance (Supplementary Table 5).

Cox univariate proportional hazard analyses demonstrated that age, sex, smoking, drinking, tumor stage, surgery history, KPS, PG-SGA, BMI, and LCR combined with CC were associated with OS (Table 3). After adjusting for age, sex, smoking, drinking, tumor stage, surgery, KPS, BMI, and PG-SGA, multivariate analysis identified low LCR combined with low CC as an adverse prognostic factor affecting the survival of patients with non-metastatic cancer (HR: 2.26; 95% CI: 1.80, 2.83; P < 0.001, Table 3). In a sensitivity analysis excluding patients who died within six months of enrollment, low LCR combined with low CC remained an adverse prognostic factor for survival in patients with non-metastatic cancer (Supplementary Table 6).

**Table 3:** Cox proportional analysis of LCR and CC predict OS in patients with non-metastatic tumors.

Variables	Univariate analysis		Multivariate analysis	
	HR (95% CI)	P-value	HR (95% CI)	P-value
Age, years				
< 65	Reference			
≥ 65	1.53 (1.32,1.77)	< 0.001		
Gender				
Male	Reference		Reference	
Female	0.51 (0.43,0.59)	< 0.001	0.76 (0.62, 0.92)	0.005
Smoking				
No	Reference			
Yes	1.77 (1.54,2.05)	< 0.001		
Drinking				
No	Reference			
Yes	1.50 (1.28,1.75)	< 0.001		
TNM				
I and II	Reference		Reference	
III	2.34 (2.01,2.72)	< 0.001	1.84 (1.58, 2.15)	< 0.001
Surgery				
No	Reference		Reference	
Yes	0.39 (0.34,0.46)	< 0.001	0.49 (0.42, 0.57)	< 0.001
Radiotherapy				
No	Reference			
Yes	0.92 (0.73,1.17)	0.508		
Chemotherapy				
No	Reference			
Yes	1.02 (0.88,1.18)	0.840		
KPS				
> 70	Reference		Reference	
≤ 70	2.41 (1.99,2.91)	< 0.001	1.49 (1.21, 1.84)	< 0.001
BMI, kg/m^2^				
< 18.5	Reference		Reference	
≥ 18.5	0.57 (0.46,0.69)	< 0.001	0.79 (0.64, 0.98)	0.034
PG-SGA				
1–3	Reference		Reference	
4–9	1.8 (1.52,2.12)	< 0.001	1.45 (1.22, 1.72)	< 0.001
> 9	2.67 (2.21,3.22)	< 0.001	1.57 (1.26, 1.94)	< 0.001
LCR CC				
LCR high and CC high	Reference		Reference	
LCR low and CC high	2.60 (2.11,3.19)	< 0.001	1.96 (1.59, 2.42)	< 0.001
LCR high and CC low	1.97 (1.62,2.39)	< 0.001	1.31 (1.06, 1.62)	0.011
LCR low and CC low	4.19 (3.44,5.1)	< 0.001	2.26 (1.80, 2.83)	< 0.001

Adjusted by age, sex, smoking, drinking, tumor stage, surgery, KPS, BMI, and PGSGA.

LCR, lymphocyte-to-C-reactive protein (CRP) ratio; CC, calf circumference; BMI, body mass index; HR, hazard ratio; CI, confidence interval.

Among the 2,797 eligible patients, 15.02% had low LCRs and CCs. The K-M curve showed that patients with low LCR and CC values had the lowest survival time, while patients with high LCR and CC values had the longest survival time (log-rank P < 0.020, Fig. 2C). After dividing the non-metastatic patients into early stage and stage III patients, the prognoses of patients with low levels of each putative biomarker were statistically significantly worse as compared with that of the other groups (log-rank P < 0.020, Fig. 2D, E).

Non-metastatic cancer patients with high LCR and CC values had shorter lengths of hospital stay (LOS) and higher Cartesian scores (KPS); these differences were statistically significant. However, LCR and CC values had no statistically significant correlations with costs (Supplementary Table 7).

### Subgroup analyses of potential confounders

To clarify the potential impact of LCR combined with CC on patient outcomes more comprehensively, we performed subgroup analysis based on several potential confounders. The results showed that low LCR combined with low CC could be used as an independent risk factor for predicting prognoses in stage I-III patients of different ages and presenting with different tumor stages, tumor types (upper gastrointestinal cancer, colorectal cancer, lung cancer), and surgery histories (Table 4).

**Table 4:** Subgroup analysis of survival in patients with non-metastatic tumor based on LCR and CC.

Variables	Univariate analysis		Multivariate analysis	
	HR (95% CI)	P-value	HR (95% CI)	P-value
TNM stage I and II tumors				
LCR high and CC high	Reference		Reference	
LCR low and CC high	2.43 (1.69,3.50)	< 0.001	2.07 (1.43, 2.99)	< 0.001
LCR high and CC low	2.54 (1.86,3.48)	< 0.001	1.86 (1.35, 2.58)	< 0.001
LCR low and CC low	4.39 (3.13,6.14)	< 0.001	2.70 (1.90, 3.85)	< 0.001
TNM stage III tumors				
LCR high and CC high	Reference		Reference	
LCR low and CC high	2.3 (1.79,2.95)	< 0.001	1.77 (1.38, 2.29)	< 0.001
LCR high and CC low	1.48 (1.15,1.89)	0.002	1.33 (1.02, 1.72)	0.033
LCR low and CC low	3.58 (2.81,4.56)	< 0.001	2.68 (2.07, 3.46)	< 0.001
Age < 65 years				
LCR high and CC high	Reference		Reference	
LCR low and CC high	2.36 (1.84,3.03)	< 0.001	1.73 (1.34, 2.22)	< 0.001
LCR high and CC low	1.72 (1.35,2.20)	< 0.001	1.38 (1.06, 1.78)	0.016
LCR low and CC low	3.89 (2.99,5.06)	< 0.001	2.46 (1.87, 3.24)	< 0.001
Age ≥ 65 years				
LCR high and CC high	Reference		Reference	
LCR low and CC high	3.17 (2.18,4.59)	< 0.001	2.48 (1.69, 3.63)	< 0.001
LCR high and CC low	2.33 (1.66,3.27)	< 0.001	1.95 (1.37, 2.76)	< 0.001
LCR low and CC low	4.30 (3.10,5.97)	< 0.001	3.38 (2.42, 4.73)	< 0.001
Upper gastrointestinal cancer				
LCR high and CC high	Reference		Reference	
LCR low and CC high	2.11 (1.35,3.31)	0.001	1.97 (1.25, 3.10)	0.004
LCR high and CC low	1.67 (1.15,2.41)	0.007	1.68 (1.14, 2.48)	0.009
LCR low and CC low	3.55 (2.43,5.18)	< 0.001	3.27 (2.20, 4.86)	< 0.001
Colorectal cancer				
LCR high and CC high	Reference		Reference	
LCR low and CC high	2.15 (1.07,4.32)	0.032	2.16 (1.06, 4.4)	0.034
LCR high and CC low	1.31 (0.71,2.41)	0.381	1.36 (0.71, 2.62)	0.358
LCR low and CC low	3.75 (2.06,6.84)	< 0.001	3.76 (1.95, 7.25)	< 0.001
Lung cancer				
LCR high and CC high	Reference		Reference	
LCR low and CC high	1.91 (1.42,2.56)	< 0.001	1.45 (1.07, 1.95)	0.015
LCR high and CC low	1.70 (1.26,2.28)	< 0.001	1.43 (1.05, 1.94)	0.022
LCR low and CC low	2.68 (2.00,3.59)	< 0.001	2.10 (1.55, 2.86)	< 0.001
Surgery				
LCR high and CC high	Reference		Reference	
LCR low and CC high	2.51 (1.79,3.50)	< 0.001	2.02 (1.44, 2.83)	< 0.001
LCR high and CC low	2.32 (1.74,3.09)	< 0.001	1.54 (1.13, 2.10)	0.006
LCR low and CC low	4.50 (3.29,6.14)	< 0.001	2.68 (1.93, 3.72)	< 0.001

LCR, lymphocyte-to-C-reactive protein (CRP) ratio; CC, calf circumference; HR, hazard ratio; CI, confidence interval.

### Prognostic value verification

Compared with the prognostic ability of LCR or CC alone, LCR combined with CC has a stronger ability to predict the prognosis of patients with non-metastatic cancer (Supplementary Fig. 5).

## Discussion

Accurately predicting prognoses for patients with non-metastatic cancers is very important for clinicians. Previous studies have shown that biomarkers of systemic inflammation are considered cancer markers and are cost-effective prognostic factors^6,26^. However, the optimal profile of systemic inflammation biomarkers and anthropometric indicators for predicting prognoses in patients with non-metastatic cancer remains unclear. A total of 2,797 patients with TNM stage I-III tumors were enrolled in this study. We first assessed the prognostic power of 13 inflammatory nutrition-associated indicators and five anthropometric indicators in this population. We found that LCR alone was a relatively good predictor of patient outcomes. Subsequently, we analyzed the synergistic effects of LCR and CC and found that patients with low LCR and CC had the worst prognoses, closely followed by prognoses when one of the indicators was high; patients with high LCR and CC values had the best prognoses. Multivariate analyses showed that low LCR combined with low CC values was independent risk factors for OS in patients with non-metastatic cancer.

A single-center prospective cohort study showed that the LCR can be used as a biomarker for predicting prognoses in patients with non-metastatic colorectal cancer^27^. He et al. found that LCR is a valuable biomarker for survival in patients with lung cancer^28^. Other studies have shown that LCR is associated with the immune status of the tumor microenvironment and can be used as a prognostic indicator for patients with liver cancer^29^. These results are consistent with our findings, and both studies showed that the LCR can be a useful biomarker for predicting prognoses in various cancers. Our study was innovative in that it enrolled a much larger sample size, evaluated a variety of cancers, and was the first to explore the relationship between LCR values and prognoses in patients with non-metastatic cancer.

LCR is directly related to lymphocyte and CRP levels. Lymphocytes are the key cells in the host cytotoxic immune response and play a vital role in the cell-mediated anti-tumor microenvironment^30,31^. Tumor-infiltrating lymphopenia is considered a predictor of poor host anti-tumor immunity and of poor prognoses^32^. CRP is an acute reactive protein regulated by interleukin-6^33^, and is a clinically recognized marker of inflammatory response.

Hart et al. found that CRP is closely associated with disease severity in cancer patients^34^. The growth of tumor cells stimulates the host to secrete interleukin-6 and other inflammatory factors, thereby increasing the synthesis of CRP in the liver^35,36^. Based on the above findings as well as the new findings of our research, we conclude that the LCR may reflect the immune status of the body as well as the systemic inflammatory response in a range of populations. Low LCR represents an impaired immune response and/or an enhanced systemic inflammatory response in cancer patients, leading to tumor progression and worse prognoses.

Abbass et al. found that inflammation plays a role in the loss of muscle mass, strength, and muscle function in cancer patients^21^. In addition, muscle loss is a diagnostic criterion for cancer cachexia^37^. CC is a simple, non-invasive, and practical indicator that can replace muscle mass. Studies have shown that CC can predict the nutritional status of hospitalized patients as well as the risk of death^38^. Our research combined LCR and CC values to concurrently evaluate inflammation levels and nutritional status. Both biomarkers are easy to measure, commonly used, safe, and have strong stability and repeatability.

In Cox multivariate analyses, LCR combined with CC was an independent prognostic factor for patients with non-metastatic cancer. Sex, TNM stage, surgery, KPS scores, BMI, and PG-SGA also independently predicted patient prognoses. Prognoses for male patients were statistically significantly worse than that of females. This may be associated with differentially expressed or Y-linked genes in males as well as unhealthy lifestyle habits such as smoking^39^. TNM staging, KPS scores, and PG-SGA scores are recognized as indicators for evaluating the condition of cancer patients and are statistically significantly related to prognosis^40–42^. Surgery is currently the preferred treatment for most patients with stage I-III tumors and can greatly improve patient prognoses. BMI is an anthropometric indicator that can reflect the nutritional status of patients with cancer and is independently associated with patient mortality^43^.

A strength of this study is that this is a multicenter observational study with a large enrolled sample size and is more representative of the overall population of patients with non-metastatic cancer as compared with prior research. This study had several limitations. First, due to its retrospective design, this study may be subject to selection bias. Second, the optimal thresholds for the LCR and CC have not yet been unified, and different datasets may lead to the determinaiton of different cut-off values. Third, the enrolled study participants were all Chinese, and the generalizabilty of our finding needs to be verified. Fourth, although this study adjusted for potential confounders, there are still certain factors that have not been considered. In addition, some of the currently known somatic mutations and molecular markers (TP53, EGFR, HER2 mutations) are poor prognostic factors, which have not been collected in our data and therefore need to be further investigated.

## Conclusions

In conclusion, our study showed that low LCR levels were statistically significantly associated with poor survival outcomes in patients with non-metastatic cancer and that this biomarker was more effective than the 12 other evaluated inflammatory markers in terms of predicting accuracy. The combined analyses of LCR and CC as potential biomarkers of overall survival provide a new basis for patients to assess prognoses. It is helpful for clinicians to classify patients according to their immune inflammatory nutritional status and to develop treatment and follow-up plans for patients accordingly. Our results thus inform research directions and will ultimately inform medical guidelines.

## Supplementary Information

Supplementary Information 1.

## Data Availability

The datasets generated and/or analysed during the current study are not publicly available because they are private databases but are available from the corresponding author on reasonable request.

## Abbreviations

AGR: Albumin–globulin ratio
AJCC: American Joint Committee on Cancer
ALI: Advanced lung cancer inflammation index
AUC: Area under the time-dependent curve
CC: Calf circumference
CI: Confidence interval
CONUT: Controlling nutritional status score
CRP: C-reactive protein
GLR: Glucose to lymphocyte ratio
GNRI: Geriatric nutritional risk index
Hb: Hemoglobin
HGS: Hand grip strength
HR: Hazard ratio
INSCOC: Chinese Common Cancer
IQR: Interquartile range
K-M curve: Kaplan-Meier curve
KPS: Karnofsky performance status
LCR: Lymphocyte-to-C-reactive protein ratio
LCS: Lymphocyte-c-reactive protein ratio score
LOS: Length of stay
MAC: Mid-arm circumference
MAMC: Mid-arm muscle circumference
mGPS: Modified Glasgow prognostic score
NLR: Neutrophil to lymphocyte ratio
NRI: Nutritional risk index
PG-SGA: Patient-generated subjective nutritional assessment
PLR: Platelet to lymphocyte ratio
PLT: Platelets
PNI: Prognostic nutritional index
ROC curve: Receiver operating characteristic curve
QLQ-C30: Core Quality of Life questionnaire
SII: Systemic Immune-Inflammation Index
TSF: Triceps skinfold thickness
