# Supplementary material for: Lymphocyte-C-reactive protein ratio with calf circumference could better predict survival of patients with non-metastatic cancer
Source: Sci Rep. 2023 May 3;13:7217. doi: 10.1038/s41598-023-34096-w (PMC10156854; doi:10.1038/s41598-023-34096-w)
Supplement: Supplementary file 1 — Supplementary Information 1. [file 41598_2023_34096_MOESM1_ESM.docx]

Supplementary Table 1. Calculation methods of 15 indicators.

| **Indicators** | **Score** | **Calculation method** |
| --- | --- | --- |
| **PNI** |  | serum levels of albumin (g/L) + 5 × absolute lymphocyte count (10^9/L) |
| **GNRI** |  | 1.489×albumin (g/L) +41.7× (present/ideal body weight) |
| **NRI** |  | [1.519×serum albumin concentration (g/L)] + [41.7× (present/ideal body weight))] |
| **NLR** |  | Nuetrophil/lymphocyte Ratio |
| **PLR** |  | Platelet/lymphocyte Ratio |
| **GLR** |  | Glucose /lymphocyte Ratio |
| **ALI** |  | 0.1×BMI×albumin (g/L)/NLR |
| **SII** |  | Nuetrophil× platelet/lymphocyte Ratio |
| **LCR** |  | 10,000× lymphocytes (10^9/L)/CRP (mg/L) |
| **AGR** |  | Albumin/globulin Ratio |
| **mGPS** | 0 | CRP ≤ 10mg/L and albumin ≥ 35g/L |
|  | 0 | CRP ≤ 10mg/L and albumin < 35g/L |
|  | 1 | CRP > 10mg/L |
|  | 2 | CRP > 10mg/L and albumin < 35g/L |
| **LCS** | 0 | Lymphocyte count ≥ 1× 10^9 /L and CRP ≤ 3mg/L |
|  | 1 | Lymphocyte count < 1× 10^9 /L and CRP ≤ 3mg/L |
|  | 1 | Lymphocyte count ≥ 1× 10^9 /L and CRP > 3mg/L |
|  | 2 | Lymphocyte count < 1× 10^9 /L and CRP > 3mg/L |
| **CONUT** | 0-1 | Albumin(g/dL): ≥3.50(0), 3.00-3.49(2), 2.50-2.99(4), <2.50(6).  Total lymphocyte count (/ml): >1,600(0), 1,200-1599(1), 800-1,199(2), <800(3).  Total cholesterol (mg/dL): >180(0), 140-180(1), 100-139(2), <100(3) |
|  | 2-4 |  |
|  | 5-8 |  |
|  | 9-12 |  |

Supplementary Table 2. C-index of 15 Inflammatory nutritional parameters and 5 anthropometric indicators.

|  | **c-index** |
| --- | --- |
| **LCR** | 0.65 |
| **NRI** | 0.64 |
| **GNRI** | 0.64 |
| **ALI** | 0.64 |
| **PNI** | 0.63 |
| **NLR** | 0.61 |
| **CONUT** | 0.61 |
| **LCS** | 0.60 |
| **mGPS** | 0.60 |
| **SII** | 0.60 |
| **AGR** | 0.60 |
| **CC** | 0.58 |
| **MAMC** | 0.57 |
| **MAC** | 0.57 |
| **GLR** | 0.57 |
| **PLR** | 0.57 |
| **HGS** | 0.56 |
| **TSF** | 0.51 |

Supplementary Table 3. Cox proportional analysis of inflammatory nutritional indicators predicts all-cause mortality in patients with non-metastatic tumor

|  | | **crude HR (95%CI)** | | ***P*-value** | | **adjusted HR (95% CI) ^a^** | | ***P*-value** | | **adjusted HR (95% CI) ^b^** | | | ***P*-value** | |
| --- | --- | --- | --- | --- | --- | --- | --- | --- | --- | --- | --- | --- | --- | --- |
| **AGR** | |  | |  | |  | |  | |  | | |  | |
| **≤1.08** | | Ref. | |  | | Ref. | |  | | Ref. | | |  | |
| **>1.08** | | 0.4 (0.34,0.48) | | <0.001 | | 0.47 (0.4,0.56) | | <0.001 | | 0.56 (0.47,0.67) | | | <0.001 | |
| **ALI, as continuous** | | 0.69 (0.63,0.75) | | <0.001 | | 0.76 (0.7,0.84) | | <0.001 | | 0.82 (0.75,0.9) | | | <0.001 | |
| **≤23.89** | | Ref. | |  | | Ref. | |  | | Ref. |  | |  |  |
| **>23.89** | | 0.41 (0.35,0.47) | | <0.001 | | 0.48 (0.41,0.56) | | <0.001 | | 0.59 (0.5,0.7) | | | <0.001 | |
| **CONUT** | |  | |  | |  | |  | |  | | |  | |
| **0-1** | | Ref. | |  | | Ref. | |  | | Ref. | | |  | |
| **2-4** | | 1.44 (1.2,1.74) | | <0.001 | | 1.36 (1.13,1.64) | | 0.001 | | 1.36 (1.13,1.64) | | | 0.001 | |
| **5-9** | | 2.25 (1.76,2.88) | | <0.001 | | 1.84 (1.43,2.37) | | <0.001 | | 1.61 (1.24,2.09) | | | <0.001 | |
| **>9** | | 3.42 (1.81,6.48) | | <0.001 | | 2.97 (1.57,5.64) | | 0.001 | | 2.65 (1.39,5.06) | | | 0.003 | |
| **GLR, as continuous** | | 1.33 (0.86,2.06) | | 0.205 | | 1.08 (0.68,1.72) | | 0.732 | | 1.24 (0.77,2) | | | 0.368 | |
| **≤4.65** | | Ref. | |  | | Ref. | |  | | Ref. | | |  | |
| **>4.65** | | 1.64 (1.41,1.91) | | <0.001 | | 1.49 (1.28,1.73) | | <0.001 | | 1.39 (1.19,1.63) | | | <0.001 | |
| **GNRI, as continuous** | | 0.69 (0.65,0.74) | | <0.001 | | 0.75 (0.7,0.81) | | <0.001 | | 0.82 (0.76,0.88) | | | <0.001 | |
| **≤97.26** | | Ref. | |  | | Ref. | |  | | Ref. | | |  | |
| **>97.26** | | 0.48 (0.41,0.55) | | <0.001 | | 0.57 (0.48,0.66) | | <0.001 | | 0.62 (0.53,0.73) | | | <0.001 | |
| **LCS** | |  | |  | |  | |  | |  | | |  | |
| **0** | | Ref. | |  | | Ref. | |  | | Ref. | | |  | |
| **1** | | 1.95 (1.64,2.32) | | <0.001 | | 1.67 (1.41,1.99) | | <0.001 | | 1.54 (1.29,1.84) | | | <0.001 | |
| **2** | | 3.24 (2.54,4.13) | | <0.001 | | 2.47 (1.93,3.15) | | <0.001 | | 2.07 (1.61,2.68) | | | <0.001 | |
| **mGPS** | |  | |  | |  | |  | |  | | |  | |
| **0** | | Ref. | |  | | Ref. | |  | | Ref. | | |  | |
| **1** | | 2.17 (1.82,2.59) | | <0.001 | | 1.94 (1.62,2.32) | | <0.001 | | 1.64 (1.37,1.97) | | | <0.001 | |
| **2** | | 2.89 (2.33,3.58) | | <0.001 | | 2.22 (1.78,2.76) | | <0.001 | | 1.57 (1.24,1.98) | | | <0.001 | |
| **NLR, as continuous** | | 1.16 (1.08,1.25) | | <0.001 | | 1.11 (1.03,1.21) | | 0.008 | | 1.09 (0.99,1.19) | | | 0.077 | |
| **≤3.39** | | Ref. | |  | | Ref. | |  | | Ref. | | |  | |
| **>3.39** | | 2.16 (1.86,2.5) | | <0.001 | | 1.85 (1.59,2.16) | | <0.001 | | 1.59 (1.36,1.86) | | | <0.001 | |
| **NRI, as continuous** | | 0.7 (0.65,0.74) | | <0.001 | | 0.75 (0.7,0.81) | | <0.001 | | 0.82 (0.76,0.89) | | | <0.001 | |
| **≤98.36** | | Ref. | |  | | Ref. | |  | | Ref. | | |  | |
| **>98.36** | | 0.47 (0.41,0.55) | | <0.001 | | 0.57 (0.48,0.66) | | <0.001 | | 0.62 (0.53,0.73) | | | <0.001 | |
| **PLR, as continuous** | | 1.16 (1.1,1.24) | | <0.001 | | 1.14 (1.07,1.21) | | <0.001 | | 1.07 (1,1.15) | | | 0.041 | |
| **≤213.56** | | Ref. | |  | | Ref. | |  | | Ref. | | |  | |
| **>213.56** | | 1.81 (1.54,2.13) | | <0.001 | | 1.57 (1.33,1.85) | | <0.001 | | 1.36 (1.15,1.61) | | | <0.001 | |
| **PNI, as continuous** | | 0.7 (0.66,0.75) | | <0.001 | | 0.76 (0.71,0.81) | | <0.001 | | 0.8 (0.75,0.86) | | | <0.001 | |
| **≤46.10** | | Ref. | |  | | Ref. | |  | | Ref. | | |  | |
| **>46.10** | | 0.49 (0.43,0.57) | | <0.001 | | 0.58 (0.5,0.67) | | <0.001 | | 0.62 (0.54,0.73) | | | <0.001 | |
| **SII, as continuous** | | 1.2 (1.13,1.26) | | <0.001 | | 1.17 (1.1,1.24) | | <0.001 | | 1.13 (1.06,1.21) | | | <0.001 | |
| **≤924.30** | | Ref. | |  | | Ref. | |  | | Ref. | | |  | |
| **>924.30** | | 2.13 (1.82,2.49) | | <0.001 | | 1.81 (1.54,2.12) | | <0.001 | | 1.53 (1.29,1.8) | | | <0.001 | |

a: Adjusted by age, gender, tumor stage, BMI

b: Adjusted by age, gender, tumor type, tumor stage, BMI, KPS, PG-SGA, surgery, radiotherapy, chemotherapy, smoking, drinking

Supplementary Table 4. Cox proportional analysis of CC predicts overall survival in patients with non-metastatic tumors by different tumor stages.

|  | **crude HR (95%CI)** | **P-value** | **adjusted HR (95% CI) ^a^** | **P-value** | **adjusted HR (95% CI) ^b^** | **P-value** |
| --- | --- | --- | --- | --- | --- | --- |
| **TNM stage I, II and III tumors** |  |  |  |  |  |  |
| **CC, as continuous** | 0.88 (0.83,0.94) | <0.001 | 0.99 (0.91,1.08) | 0.842 |  |  |
| **≤29.8/≤34.5** | Ref. |  | Ref. |  | Ref. |  |
| **>29.8/>34.5** | 0.68 (0.59,0.79) | <0.001 | 0.84 (0.71,1) | 0.056 | 0.83 (0.69,0.99) | 0.042 |
| **p for trend** |  | <0.001 |  | 0.253 |  | 0.260 |

a: Adjusted by age, gender, tumor stage, BMI

b: Adjusted by age, gender, tumor type, tumor stage, BMI, KPS, PG-SGA, surgery, radiotherapy, chemotherapy, smoking, drinking

Abbreviations: CC, calf circumference; HR, hazard ratio; CI, confidence interval.

Supplementary Table 5. Mean survival time of different LCR combined with CC groups.

|  | **Cases** | **Mean survival months (CI)** | ***P*-value** |
| --- | --- | --- | --- |
| **Non-metastatic cancer** |  |  |  |
| **LCR high and CC high** | 1243 | 52.12(51.11,53.13) | <0.001 |
| **LCR low and CC high** | 448 | 41.17(38.86,43.48) |  |
| **LCR high and CC low** | 686 | 45.32(43.58,47.06) |  |
| **LCR low and CC low** | 420 | 34.15(31.58,36.72) |  |
| **<65years** |  |  |  |
| **LCR high and CC high** | 932 | 52.26(51.10,53.41) | <0.001 |
| **LCR low and CC high** | 312 | 42.76(40.05,45.47) |  |
| **LCR high and CC low** | 445 | 46.66(44.55,48.77) |  |
| **LCR low and CC low** | 210 | 35.90(32.13,39.66) |  |
| **≥65years** |  |  |  |
| **LCR high and CC high** | 311 | 51.75(49.66,53.83) | <0.001 |
| **LCR low and CC high** | 136 | 37.52(33.19,41.84) |  |
| **LCR high and CC low** | 241 | 42.89(39.86,45.93) |  |
| **LCR low and CC low** | 210 | 32.58(29.06,36.09) |  |
| **Male** |  |  |  |
| **LCR high and CC high** | 482 | 49.31(47.47,51.15) | <0.001 |
| **LCR low and CC high** | 202 | 38.42(34.85,41.98) |  |
| **LCR high and CC low** | 568 | 44.88(42.94,46.81) |  |
| **LCR low and CC low** | 352 | 33.13(30.27,35.99) |  |
| **Female** |  |  |  |
| **LCR high and CC high** | 761 | 53.83(52.67,55.00) | <0.001 |
| **LCR low and CC high** | 246 | 43.13(40.11,46.15) |  |
| **LCR high and CC low** | 118 | 47.42(43.51,51.32) |  |
| **LCR low and CC low** | 68 | 39.01(33.22,44.80) |  |

Supplementary Table 6. COX analysis of non-metastatic patients with survival of less than 6 months was excluded.

| **Variables** | **Univariate Analysis** | | **Multivariate Analysis** | |
| --- | --- | --- | --- | --- |
|  | **HR（95%CI）** | ***P-value*** | **HR（95%CI）** | ***P-value*** |
| **Gender** |  |  |  |  |
| Male | Reference |  |  |  |
| Female | 0.51 (0.43,0.61) | <0.001 |  |  |
| **Age，years** |  |  |  |  |
| <65 | Reference |  |  |  |
| ≥65 | 1.45 (1.23,1.70) | <0.001 |  |  |
| **Smoking** |  |  |  |  |
| No | Reference |  |  |  |
| Yes | 1.82 (1.55,2.13) | <0.001 | 1.24 (1.02,1.50) | 0.031 |
| **Drinking** |  |  |  |  |
| No | Reference |  |  |  |
| Yes | 1.46 (1.22,1.74) | <0.001 |  |  |
| **Digestive system cancer** |  |  |  |  |
| No | Reference |  |  |  |
| Yes | 0.89 (0.76,1.05) | 0.171 |  |  |
| **TNM** |  |  |  |  |
| I and II | Reference |  | Reference |  |
| III | 2.30(1.94,2.71) | <0.001 | 1.86 (1.57,2.20) | <0.001 |
| **Surgery** |  |  |  |  |
| No | Reference |  | Reference |  |
| Yes | 0.38 (0.32,0.45) | <0.001 | 0.47 (0.40,0.55) | <0.001 |
| **Radiotherapy** |  |  |  |  |
| No | Reference |  |  |  |
| Yes | 0.88 (0.68,1.15) | 0.352 |  |  |
| **Chemotherapy** |  |  |  |  |
| No | Reference |  |  |  |
| Yes | 1.04 (0.88,1.23) | 0.638 |  |  |
| **KPS** |  |  |  |  |
| >60 | Reference |  | Reference |  |
| ≤60 | 2.27 (1.63,3.16) | <0.001 | 1.45 (1.01,2.08) | 0.045 |
| **BMI, kg/m²** |  |  |  |  |
| <18.5 | Reference |  |  |  |
| 18.5-23.9 | 0.69 (0.54,0.88) | 0.003 |  |  |
| ≥24 | 0.58 (0.45,0.75) | <0.001 |  |  |
| **PG-SGA** |  |  |  |  |
| 0-3 | Reference |  | Reference |  |
| 4-9 | 1.64 (1.37,1.97) | <0.001 | 1.38 (1.15,1.66) | 0.001 |
| >9 | 2.20 (1.78,2.73) | <0.001 | 1.42 (1.12,1.81) | 0.004 |
| **LCR CC** |  |  |  |  |
| LCR high and CC high | Reference |  | Reference |  |
| LCR low and CC high | 2.19 (1.73,2.76) | <0.001 | 1.69 (1.34,2.14) | <0.001 |
| LCR high and CC low | 1.95 (1.58,2.40) | <0.001 | 1.42 (1.12,1.80) | 0.004 |
| LCR low and CC low | 3.73 (3.00,4.65) | <0.001 | 2.34 (1.80,3.04) | <0.001 |

Supplementary Table 7. Relationship between LCR, CC and secondary outcome events in patients with non-metastatic tumor

| **Outcome** | **Overall(n=2797)** | **LCR** | | | **CC** | | |
| --- | --- | --- | --- | --- | --- | --- | --- |
|  |  | **≤ 2500(n=868)** | **> 2500(n=1929)** | ***P* value** | **Male≤34.5&Female≤29.8(n=1106)** | **Male>34.5&Female>29.8(n=1691)** | ***P* value** |
| **LOS (mean (SD))** | 13.33 (12.12) | 14.23 (11.80) | 12.93 (12.24) | 0.008 | 14.19 (13.03) | 12.77 (11.45) | 0.003 |
| **COST (mean (SD))** | 28126.85 (50479.98) | 29362.43 (41802.27) | 27570.87 (53932.04) | 0.385 | 29226.73 (39061.69) | 27407.47 (56717.97) | 0.351 |
| **KPS (mean (SD))** | 87.82 (10.31) | 84.88 (12.70) | 89.14 (8.71) | <0.001 | 85.91 (11.36) | 89.07 (9.35) | <0.001 |

Supplementary Figure 1. Time-dependent ROC curves for the 13 nutritional and Inflammatory biomarkers of OS.


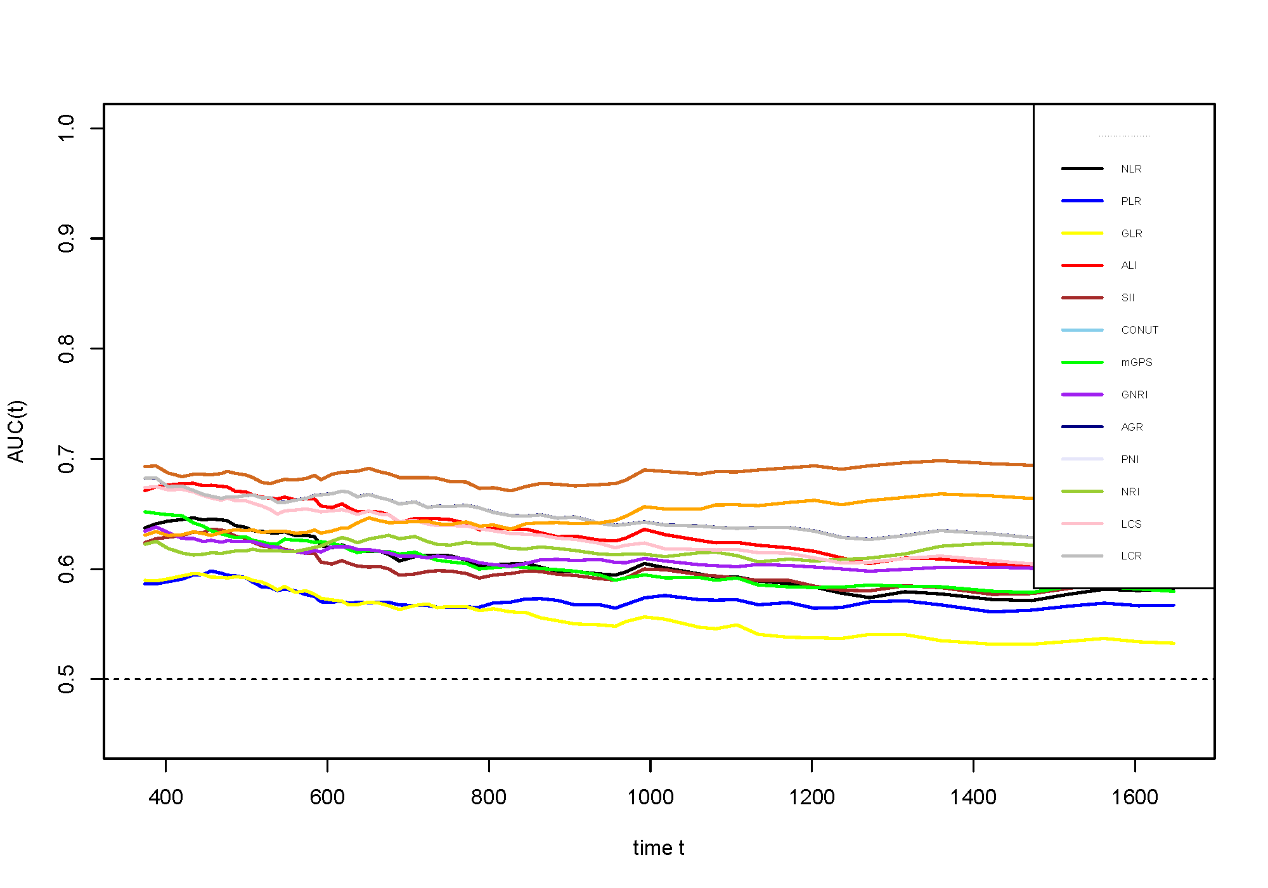


Abbreviations: NLR, neutrophil-to-lymphocyte ratio; PLR, platelet-to-lymphocyte ratio; GLR, glucose-to-lymphocyte ratio; ALI, advanced lung cancer inflammation index; SII, systemic immune-inflammation index; CONUT, controlling nutritional status score; mGPS, modified Glasgow Prognostic Score; GNRI, Geriatric Nutritional Risk Index; AGR, albumin-globulin ratio; PNI, prognostic nutritional index; NRI, nutritional risk index; LCS, lymphocyte C-reactive protein score; LCR, lymphocyte-to-C-reactive protein (CRP) ratio

Supplementary Figure 2. Time-dependent changes in the area under the curve (AUC) for survival of HGS, MAC, MAMC, TSF and CC.


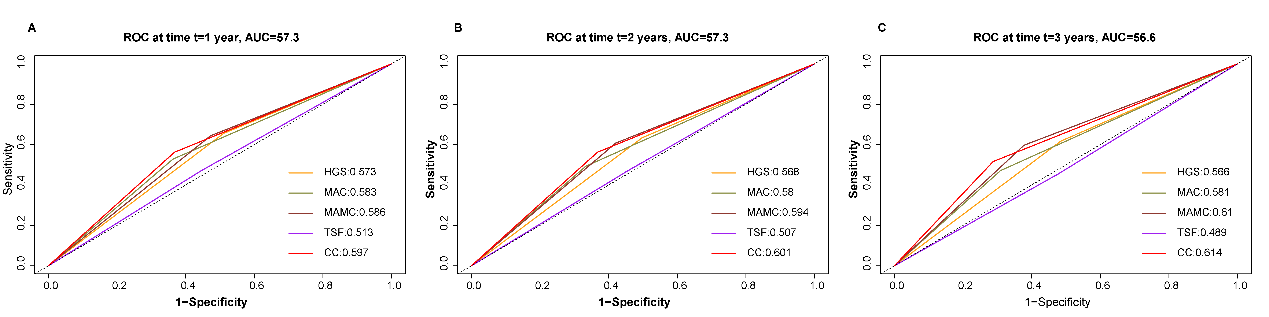


1. ROC curves for 1-year overall survival; B. ROC curves for 2-year overall survival, C. ROC curves for 3-year overall survival.

Abbreviations: ROC, receiver operating characteristic; HGS, hand grip strength; MAC, mid-arm circumference; MAMC, mid-arm muscle circumference; CC, calf circumference; TSF, triceps skinfold.

Supplementary Figure 3. Cut-off value of LCR in patients with non-metastatic tumor


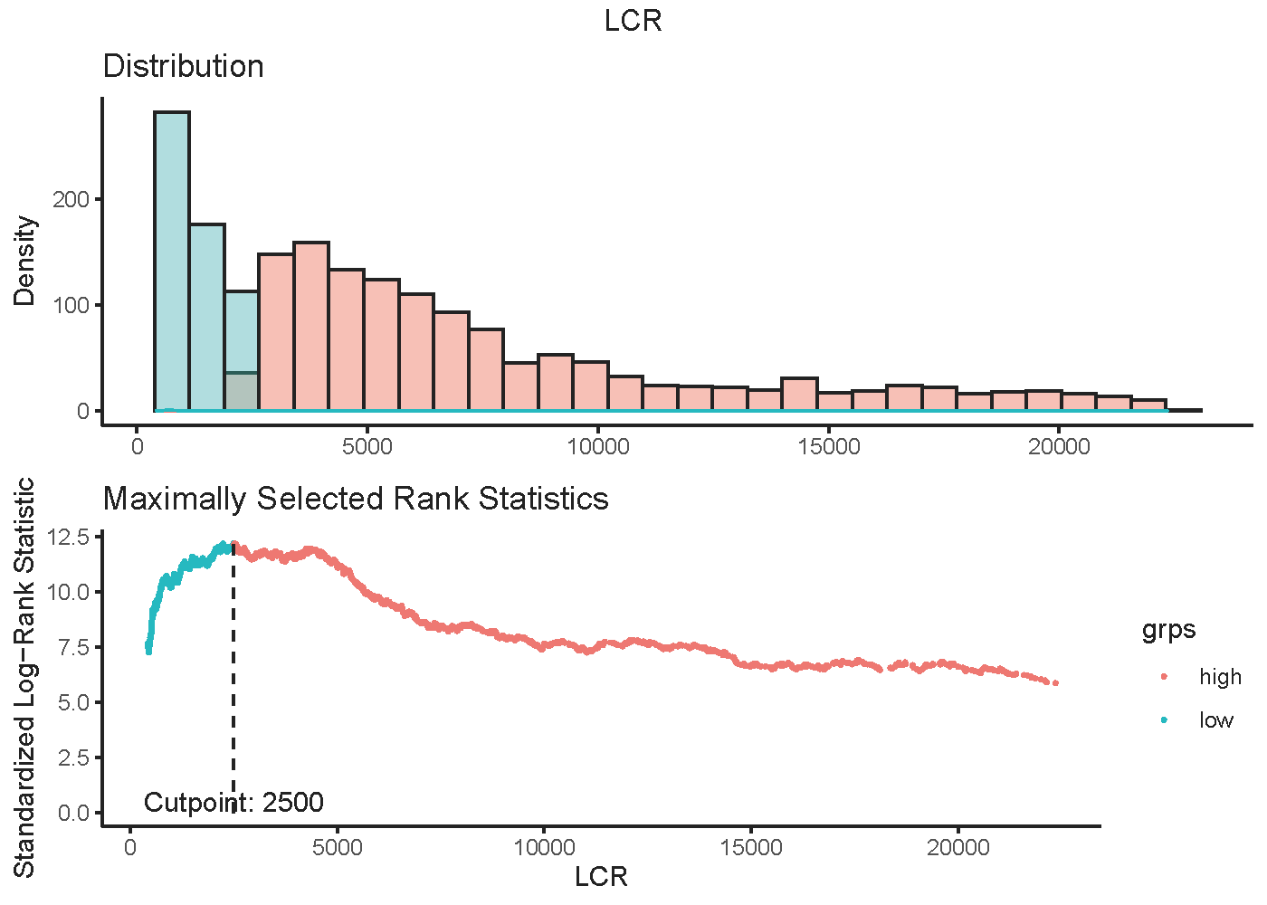


Supplementary Figure 4. The association of LCR with overall survival in patients with non-metastatic tumor.


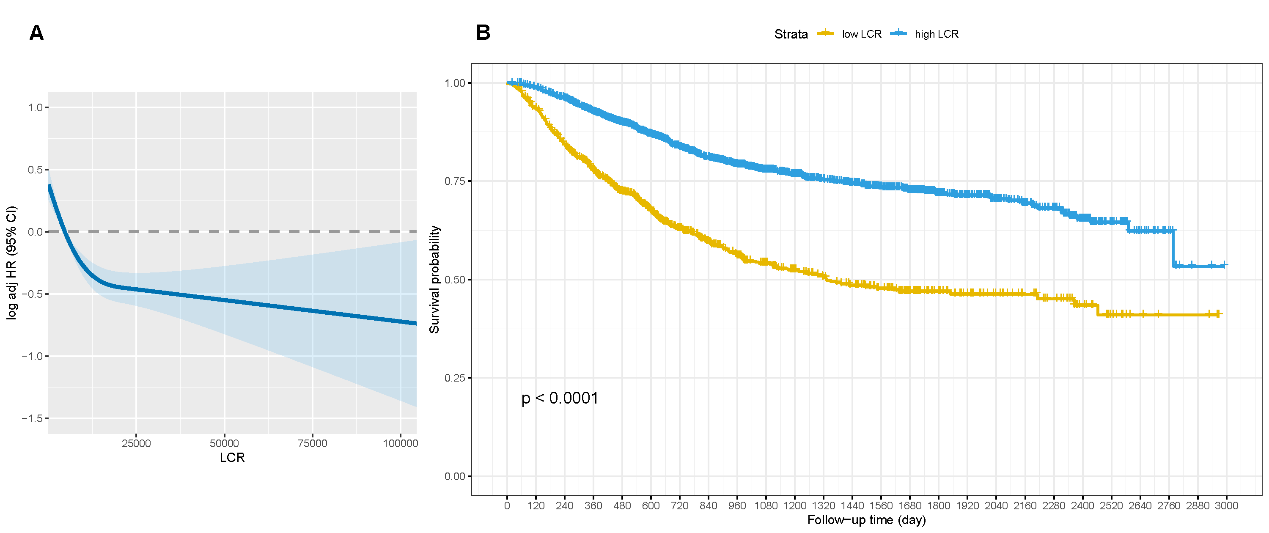
A. The overall survival is shown after adjusted for age, gender, tumor type, tumor stage, BMI, KPS, PG SGA, surgery, radiotherapy, chemotherapy, smoking, drinking. The x-axis shows the level of LCR. The curve shows the incidence, with 95% CI, of the estimates. B. Kaplan-Meier curve of LCR classification of patients with non-metastatic cancer.

Supplementary Figure 5. The prognostic value of LCR combined with CC is stronger than the two alone


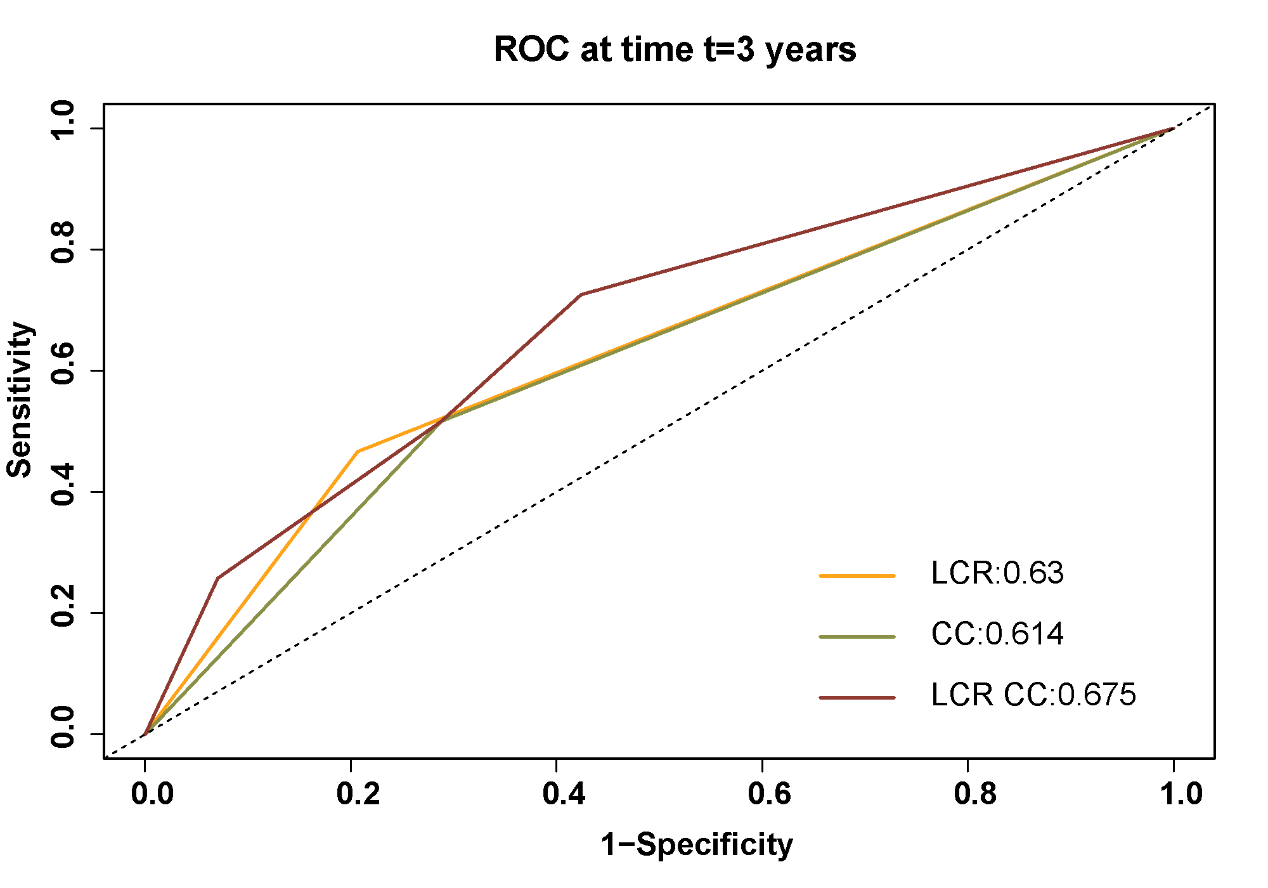


Abbreviations: ROC, receiver operating characteristic; LCR, lymphocyte-to-C-reactive protein (CRP) ratio; CC, calf circumference.
